# Exploring the Tumor-Associated Risk of Mesenchymal Stem Cell Therapy in Veterinary Medicine

**DOI:** 10.3390/ani14070994

**Published:** 2024-03-23

**Authors:** Soyoung Jeung, Sungsoo Kim, Jaegon Ah, Sanghyuk Seo, Umair Jan, Hyejin Lee, Jeong Ik Lee

**Affiliations:** 1 VIP Animal Medical Center, 73, Dongsomun-ro, Seongbuk-gu, Seoul 02830, Republic of Korea; jeungs1007@gmail.com (S.J.); ilovekh0@vipah.co.kr (S.K.); sadgon@hanmail.net (J.A.); ssh45035@vipah.co.kr (S.S.); 2 Laboratory of Veterinary Internal Medicine, Department of Veterinary Clinical Science, College of Veterinary Medicine, Seoul National University, Gwanak-ro, Gwanak-gu, Seoul 08826, Republic of Korea; 3 Regenerative Medicine Laboratory, Center for Stem Cell Research, Department of Biomedical Science and Technology, Institute of Biomedical Science and Technology, Konkuk University, Seoul 05029, Republic of Korea; umairjan47@konkuk.ac.kr; 4 Department of Veterinary Obstetrics and Theriogenology, College of Veterinary Medicine, Konkuk University, Seoul 05029, Republic of Korea; hyejinly@konkuk.ac.kr

**Keywords:** mesenchymal stem cells, tumorigenesis, canine, feline, adipose-derived MSCs

## Abstract

**Simple Summary:**

This report discusses the ongoing research on, and concerns regarding, the tumor promotion associated with multipotent stem cells, with a focus on mesenchymal stem cells (MSCs) in regenerative medicine. Throughout this commentary, we explore the various impacts of MSC therapies on tumor growth that occurs in veterinary analysis. Additionally, we investigated the characteristics that rendered embryonic stem cells and induced pluripotent stem cells more tumorigenic than MSCs. Overall, despite concerns regarding tumorigenesis, limited evidence supports the increased risk of tumors associated with MSC injections. MSCs are thus considered a valuable and safe alternative in the veterinary medicine market, especially for companion animals such as dogs and cats. Moreover, bioengineered MSC-derived exosomes are preferable choices for tumor diagnosis and treatment.

**Abstract:**

Mesenchymal stem cell (MSC) therapy has been actively applied in veterinary regenerative medicine to treat various canine and feline diseases. With increasing emphasis on safe cell-based therapies, evaluations of their tumorigenic potential are in great demand. However, a direct confirmation of whether tumors originate from stem cells or host cells is not easily achievable. Additionally, previous studies evaluating injections of high doses of MSCs into nude mice did not demonstrate tumor formation. Recent research focused on optimizing MSC-based therapies for veterinary patients, such as MSC-derived extracellular vesicles in treating different diseases. This progress also signifies a broader shift towards personalized veterinary medicine, where treatments can be tailored to individual pets based on their unique genetic profiles. These findings related to different treatments using MSCs emphasize their future potential for veterinary clinical applications. In summary, because of lower tumor-associated risk of MSCs as compared to embryonic and induced pluripotent stem cells, MSCs are considered a suitable source for treating various canine and feline diseases.

## 1. Introduction

Multipotent mesenchymal stem cells (MSCs) derived from bone marrow were first identified in 1961 [1]. Stem cells have since been actively researched for several years in both human and veterinary medicine, and commercial stem cell therapy for equine orthopedic diseases commenced in 2003 [2]. As a cornerstone of regenerative medicine, stem cell therapy has primarily been used to treat cellular damage and refractory diseases in dogs and cats [3]. However, recently, its therapeutic scope has gradually expanded, with a notable emphasis on its anti-inflammatory and immunomodulatory capabilities [4,5,6].

Stem cell therapy in dogs and cats utilizes either in-clinic cell production or the use of approved veterinary medical products. Japan introduced the first allogeneic adipose-derived MSC-based pharmaceutical (Stemcure^®^, Osaka, Japan), and the UK pioneered a xenogeneic stem cell-based pharmaceutical (DogStem^®^, Oxfordshire, UK) using equine umbilical cord-derived MSCs, primarily for use in dogs [7,8].

Safety plays a pivotal role as a prerequisite and a fundamental element in decision making related to stem cell therapy [9]. Previous research has suggested that the side effects of stem cell therapy are minimal, generally mild, and self-limiting over a short period [10]. However, the tumor-associated risk of MSCs remains uncertain; thus, to ensure safe stem cell therapy, there is a need to assess the risk of tumorigenesis and tumor promotion.

Animal model research simulates naturally occurring human diseases and is driven by the extensive parallels between animals and humans in comparative medicine [11,12,13]. Ongoing research has explored the tumorigenesis of MSCs after in-vivo injections as well as their impact on tumor growth. Moreover, some studies have demonstrated the contributions of animal models in studying human diseases, providing numerous similarities between human and companion animals [14,15,16,17,18,19,20]. Considering these aspects, in this commentary, our focus is on exploring the research on the tumor-associated risk of stem cell therapies in companion animals, as based on insights from human biology and medicine. The aim of this study is to thoroughly assess the safety of MSC therapy by examining it from multiple perspectives. This involves analyzing its distinct characteristics and comparing its safety profile with that of embryonic stem cells and induced pluripotent stem cells, as well as the risk associated with tumors. Additionally, this study seeks to explore how MSCs can be effectively applied in stem cell therapy.

## 2. Assessing the In Vivo Tumor-Associated Risk of MSCs: A Potential Area of Concern?

The potential tumor promotion of MSCs during in vivo use has been a subject of discussion in the fields of regenerative and veterinary medicine. The mechanisms underlying tumor promotion are diverse and complex, however; thus, investigating the role of MSC therapy in tumor development is a complex task. In practice, distinguishing whether an emerging tumor originated from stem cells or the patient’s own cells is exceedingly challenging. It is difficult to predict how stem cells will interact with various cytokines and signaling pathways in vivo, primarily because in vivo conditions differ substantially from in vitro settings.

Immune system weakening can theoretically increase the susceptibility to tumor development [21]. Several studies have investigated stem cell tumorigenicity in immunodeficient mice. In NOD/SCID mice, canine adipose-derived stem cells were subcutaneously injected, and after a 40 d observation period, no tumor formation was observed in contrast to the positive control group, which received HeLa cells [22]. Similarly, in nude mice, various doses of human adipose-derived MSCs were injected and monitored for 13 weeks after intravenous injection and 26 weeks after subcutaneous injection. In all dose groups, including the high-dose group, no toxicities or tumorigenesis instances were observed [23]. Additionally, canine adipose-derived MSCs were injected into 30 balb/c-nu mice for a safety and tumorigenesis evaluation of MSCs and monitored for 6 months. There were no tumorigenesis cases in the treatment group, whereas all mice in the control group that had been injected with A-431, an epidermoid carcinoma cell line, showed tumorigenesis [24]. A research reported that human adipose tissue-derived mesenchymal stem cells inhibit the growth of T-cell lymphoma by switching cell-cycle progression and inducing apoptosis in nude mice [25]. Although these results suggest that direct tumorigenicity from MSCs through subcutaneous and systemic injections is unlikely, concerns regarding the sensitivity of in vivo tumorigenicity assays remain [26]. Therefore, it is crucial to further scrutinize these results and explore the remaining key factors that may influence the reliability of such analyses in predicting tumorigenic outcomes, as taking these steps can ensure a comprehensive evaluation of the potential risks associated with stem cell therapies and address any existing uncertainties in their safety profiles.

## 3. Harnessing the Dual Nature of MSCs: Tumor Promotion Implication and Cancer Treatment

MSCs have a dual nature, exhibiting both pro-tumorigenic and anti-tumorigenic effects. Understanding these mechanisms is crucial to assessing the risks associated with MSC therapy in veterinary oncology. Tumorigenesis involves the entire tumor formation process, including the initiation, promotion, and progression stages. Tumor promotion refers to the stage in which initiated cells are stimulated to proliferate and survive, thereby promoting their development. The pro-tumorigenic characteristic of MSCs can be attributed to various mechanisms, such as immunosuppression, creating a favorable microenvironment for tumor cells to evade immune surveillance. Moreover, MSCs can interact with tumor cells and contribute to their resistance through chemoresistance by chemotherapy agents, thereby negatively impacting the effectiveness of treatments [27]. The promotion of angiogenesis is also crucial for tumor growth, as it ensures a sufficient blood supply to the tumor, aiding in its sustenance and expansion and enhancing tumor cell survival and proliferation [28]. MSCs can also undergo a phenotypic transition to become tumor-associated fibroblasts in response to secreted growth factors and extracellular matrix components [29,30]. Furthermore, cancer stem cell correlation, metastasis facilitation, cancer cell apoptosis inhibition, and extracellular signal-regulated kinase 1/2 (ERK1/2) pathway activation collectively support MSC tumor growth through an epithelial–mesenchymal transition [31,32].

In contrast, MSCs can also be used in cancer treatments. Brain tumors, characterized by a high fatality rate, are notoriously challenging for delivering anti-cancer agents because of the blood–brain barrier (BBB) [31]. However, MSCs can act as a ‘Trojan horse;’ through this mechanism, they allow the delivery of cytotoxic compounds to brain tumor cells, offering an anti-tumor treatment to patients with brain tumors [33]. Han et al. demonstrated that engineered MSCs can exert anti-cancer properties. IFN-β-transduced canine adipose tissue-derived MSCs inhibited the growth of canine melanoma cells in an in vitro direct/indirect co-culture system [34,35]. Furthermore, in vivo studies incorporating BALB/c nude mouse xenografts have revealed that combining these engineered MSCs with chemotherapy enhances their anti-tumor efficacy [35]. MSCs inhibit tumor growth via mechanisms such as cell cycle arrest, inflammatory cell infiltration, cancer cell apoptosis, and regulation of WNT/AKT signaling [31].

## 4. Exploring Boundless Potential: MSC Therapy Unveils Promising Applications

Recent advances in regenerative medicine have prompted a paradigm shift from cell-based therapies to cell-free therapies using MSC-derived substances [36,37]. Extracellular vesicles, particularly exosomes, which are derived from MSCs and are a small member vesicle, play a pivotal role in intercellular communication by transferring bioactive molecules. The use of MSC-derived exosomes has emerged as a promising approach for therapeutic interventions, and several aspects noted in previous studies highlight the importance of exosomes in regenerative medicines [38].

The administration of MSC-derived exosomes can exert divergent dual effects on tumor growth. MSC-derived cytokines or exosomes can promote tumor growth via multiple mechanisms. Conversely, MSC-derived exosomes, which contain specific miRNAs, have shown promise in facilitating cancer screening, diagnosis, and early detection [39]. 

Conventional tumor diagnosis primarily relies on procedures such as fine-needle aspiration for cytology and tissue biopsies for histology [40]. However, these approaches have limitations owing to the small scope of the information they offer and their invasive nature. In contrast, exosomes offer convenient liquid biopsy-based testing and can serve as both diagnostic and prognostic biomarkers [41]. Detecting tumor-related miRNAs within exosomes facilitates cancer screening, diagnosis, and early detection [42,43,44,45,46]. Specific miRNAs are associated with tumor treatment prognosis in the TNM (tumor, node, and metastasis) stage and resistance to radiation therapy [47,48]. Exosomes exhibit an excellent BBB permeability and lower immunogenicity than stem cells do. Thus, the former are promising therapeutic agents. MSC-derived exosomes can serve as effective drug carriers for targeted cancer therapies [49]. MSC-derived nanoparticles contain specific miRNAs that can inhibit angiogenesis, migration, and metastasis in glioblastomas [35]. Thus, researchers are actively studying the use of engineered MSC exosomes to address the two-sided effects of exosomes on tumor growth. They are also advancing tumor treatments by developing exosome mimetics to overcome the limitations of small-scale natural exosomes.

## 5. Why Do Embryonic Stem Cells (ESCs) and Induced Pluripotent Stem Cells (iPSCs) Have a Higher Oncogenic Risk Than MSCs?

Multipotent MSCs, being distinct from pluripotent stem cells such as ESCs and iPSCs, exhibit a more limited differentiation capacity [50]. These stem cells can differentiate into chondrogenic, adipogenic, and osteogenic lineages and maintain a high ability for self-renewal [51]. Controlled differentiation with multipotency is a crucial aspect for therapeutic applications, as it provides researchers and clinicians a means to guide the cells towards desired lineages. In controlled differentiation, the MSCs are directly differentiated into specific cell types under controlled conditions. This characteristic makes multipotent stem cells a promising tool for regenerative therapies in veterinary medicine [52].

In contrast, pluripotent stem cells can differentiate into all three germ layers as well as all types of cells exhibiting unlimited proliferation [53]. Similarly, no major health concerns were reported in small-sized animals for clinical trials conducted using MSCs in regenerative medicine fields, which means that MSC treatments are relatively safer than pluripotent stem cell treatments since they have a higher rate of proliferation [30]. These undifferentiated pluripotent stem cells can contribute to tumor formation, both benign and malignant, in immunodeficient animal models via continued proliferation after an in vivo transplantation.

The reprogramming methods for iPSCs are broadly classified into viral and non-viral methods [54]. The initial iPSCs were induced from adult-derived cells through retroviral transfection, incorporating four reprogramming factors: Oct3/4, Sox2, Klf4, and cMyc. These factors reprogram somatic cells into cells with ESC-like conditions [55]. These are also the main pluripotency factors in ESCs [56] and are closely associated with tumorigenesis. Pluripotency factors such as Sox2 and Klf-4 in ESCs and iPSCs promote tumorigenesis [57,58]. Sox2 can ensure tumor cell survival and is oncogenic in several kinds of cancers of the lung and esophagus, such as carcinomas [59]. In the context of MSC therapies, it has been documented that the reactivation of the c-myc oncogene can contribute to tumor formation. Similarly, the factor Klf-4, which promotes cellular proliferation in breast cancer by suppressing P53, a tumor suppressor, also plays a role in suppression. Therefore, these pluripotency-related transcription factors, including c-myc, can potentially induce oncogenesis and malignancies, emphasizing the necessity of comprehensive safety assessments in MSC-based therapies [58,59,60].

Furthermore, pluripotent stem cells can undergo genetic alterations—chromosome abnormalities, variations in copy number, and single nucleotide mutations—during in vitro experimentations [61]. Although the likelihood of a single genetic mutation causing tumorigenesis is low, an accumulation of mutations in tumor-related genes increases the risk of tumorigenesis [62]. Thus, concerns regarding the tumorigenesis of ESCs and iPSCs persist and represent a critical hurdle to patient treatment.

## 6. Conclusions

Tumorigenesis is a complex interplay between cellular genetic factors and environmental influences, with its precise etiology and mechanisms yet to be fully elucidated. Given the proliferative characteristics of stem cells, concerns regarding the tumor-associated risk of MSCs remain unresolved. This report aimed to address the increasing demand for safety in the expanding market of MSC therapy and to bridge the gap in our understanding of tumor promotion of stem cells.

We probed the capacity of MSCs to induce tumor formation in vivo. The prevailing consensus suggests that robust evidence supporting an increased tumor incidence associated with MSC injections is limited. Moreover, the process of distinguishing the region of tumor origin remains ambiguous. Therefore, it may be preemptive to conclude that MSC injections cause risks related to tumors, as the relationship between MSCs and tumorigenesis or tumor promotion remains unrevealed in many cases.

The dual nature of MSCs presents both challenges and opportunities in the realm of veterinary oncology. Exploring the potential of tumor promotion as well as the tumorigeneses of MSC therapy requires an understanding of the interplay between both pro-tumorigenic and anti-tumorigenic effects. Veterinary researchers and clinicians can exploit this dual nature to develop targeted and personalized approaches for cancer treatments in companion animals, thereby facilitating innovative veterinary medicine strategies. Concurrently, the use of MSC-derived exosomes in veterinary medicine presents a promising venue for therapeutic interventions, potentially aiding in early cancer detection and treatment.

Although MSCs may have limited multipotency compared to ESCs and iPSCs, they offer the distinct advantage of a lower risk of tumor promotion. MSCs can thus be considered a valuable and safe choice for future treatments of canine and feline patients.

In this commentary, we explored the tumor-associated risk of MSC therapies in veterinary medicine. The prospects of MSC therapies require a comprehensive understanding of tumor promotion, the development of safer treatment modalities, and exploration of novel applications in both human and veterinary medicine. Continued research efforts will remain vital to unlocking the full potential of MSC-based therapies while ensuring their safety and efficacy in clinical applications.

## Data Availability

Not applicable.
